# Occupational health risks and provision gaps among live-in caregivers: findings from a qualitative interview study of Polish live-ins in Germany

**DOI:** 10.1186/s12995-026-00522-8

**Published:** 2026-08-07

**Authors:** Lea Scheuvens, Maria Girbig, Melanie Schubert, Charlyn Görres, Amelie Schirmer, Agnieszka Sykut, Stephanie Drössler, Verena Weihofen, Liv Hübner, Daniel Kämpf, Charlotte Pietzsch, Katja Schladitz, Franziska Welzel, Nadja-Raphaela Baer, Steffi Riedel-Heller, Liane Schenk, Margrit Löbner, Andreas Seidler

**Affiliations:** 1 Institute and Policlinic of Occupational and Social Medicine (IPAS), Faculty of Medicine, Technische Universität Dresden, Dresden, Germany; 2 Institute of Social Medicine, Occupational Health and Public Health (ISAP), Medical Faculty, University of Leipzig, Leipzig, Germany; 3 Institute of Medical Sociology and Rehabilitation Science, Charité – University Medical Center Berlin, Freie Universität Berlin and Humboldt-Universität zu Berlin, Berlin, Germany; 4 Institute of Psychology, Humboldt-Universität zu Berlin, Berlin, Germany

**Keywords:** Health prevention and provision, Home-based care, Live-in, Temporary migrant workers, Occupational health care, Occupational safety, Qualitative interview study

## Abstract

**Background:**

Migrant live-in caregivers play an important role in home-based long-term care, yet their working and living conditions remain largely underregulated. Despite increasing evidence of precarious employment and health risks among live-in caregivers internationally, little is known about their occupational health risks and access to occupational health care.

**Methods:**

This qualitative interview study is part of the project *Live-In Health* and reports findings from 16 semi-structured interviews with Polish live-in caregivers (15 females and one male) working in German private households. Interviews were conducted in Polish, transcribed, translated into German, and analyzed using qualitative content analysis following an inductive-deductive approach. Identified work-related strains and risks were structured in four main domains according to established occupational risk assessment frameworks from the German care sector.

**Results:**

Participants reported extensive occupational health risks across the four main domains: physical strain (e.g., musculoskeletal strain from patient transfers), psychological strain (e.g., social isolation, conflicts with agencies, emotional burden), occupational safety and health including working hours (e.g., long working hours, insufficient recovery time, lack of preparation and insurance coverage), and exposure to biological and hazardous substances (e.g., wound care, unhygienic living conditions). Access to occupational and general health care in Germany was largely absent. Preventive services, occupational medical examinations, and accident insurance coverage were inconsistently available or missing altogether. Live-ins described substantial barriers related to legal ambiguity, agency practices, lack of information, and unclear responsibilities. At the same time, informal support from families, ambulatory care services, and peer networks emerged as partial coping resources.

**Conclusions:**

Polish live-in caregivers in Germany are exposed to considerable occupational health risks while remaining largely excluded from occupational health care structures. The findings highlight substantial gaps in the implementation of existing occupational safety and health regulations for caregivers in private households. Strengthening occupational health protection for live-in caregivers requires enforceable working time regulations, reliable insurance coverage, accessible occupational health services, and clearer oversight of intermediary agencies. Tailored, low-threshold occupational health approaches are urgently needed to address the structural vulnerabilities of this workforce and to ensure healthy and sustainable care provision for the growing demand in home-based care for older people in Germany.

**Supplementary Information:**

The online version contains supplementary material available at 10.1186/s12995-026-00522-8.

## Background

Labor migration is a global phenomenon in which individuals leave their home countries to take up temporary or long-term employment abroad, primarily to improve their socio-economic situation [1, 2]. According to the International Labor Organization (ILO) estimates [13], approximately 155.6 million people were working as migrant workers worldwide in 2022 [3]. The share of migrants, employed in the care economy – including health care and domestic services – was estimated at 12.4% for men and 28.8% for women.

Germany’s long-term care system is highly dependent on this workforce. In December 2023, about 5.7 million people were considered in need of care under the German Long-Term Care Insurance Act (SGB XI), of whom about 86% (4.9 million) were cared for at home [4]. Home-based care, often supported by live-in caregivers, offers financial advantages compared to institutional settings, relieves family members, and ensures continuous availability of care – particularly important for individuals with dementia or other cognitive impairments [5–7]. Live-in caregivers typically perform a wide range of tasks, including household duties and basic personal care such as assistance with hygiene, mobility, eating, and daily routines. The private household serves simultaneously as both the workplace and the place of residence for live-in caregivers. Live-in care creates complex forms of relationships that are deeply personal yet structured by unequal global systems of migration, labor, and care [8]. 

It is estimated that between 300,000 and 700,000 migrant caregivers provide support in live-in arrangements in Germany, mostly assisting older people within their own homes [6, 9–11]. The majority of these live-in caregivers are women from Central and Eastern Europe, with Poland representing the most frequent country of origin [6, 12]. Employment is typically temporary, structured in two- to three-month rotational systems with multiple caregivers alternating over time [9, 11]. Three employment models exist: (a) the employer model, in which the household directly employs the caregiver; (b) the posting of workers model, in which a foreign company deploys the caregiver to the household under EU regulations; and (c) self-employment. The posting model, followed by self-employment, represents the most common arrangement. For live-ins, these transnational and often short-term employment relationships are frequently associated with limited labor protection and high psychological strain [13–15].

Several studies highlight the structural vulnerability of migrant live-in caregivers. Migrant workers are often disadvantaged in terms of working conditions, wages, and occupational safety, and they face a higher risk of occupational accidents than non-migrant workers [16]. A scoping review including 27 studies from English-, Chinese-, and Indonesian-speaking countries identified key stressors for migrant domestic workers, including work-related abuse and exploitation, restricted access to health care and health insurance, financial pressure despite demanding work, and social isolation [17]. Studies conducted in Germany further point to specific stressors related to live-in care arrangements, such as dealing with patients with complex psychological or cognitive conditions (e.g. dementia), a lack of rest periods, and constant availability due to the absence of a clear separation between work and private life [6, 14, 18–20]. The live-in setting also creates a high degree of dependency on employers [18, 21, 22].

Previous studies indicate major challenges regarding the occupational health and safety of live-in caregivers. For example, a survey of migrant home-care workers in Israel reported widespread violations of labor rights, including the lack of paid sick leave (79% of respondents) and limited vacation time; conditions that may negatively affect health, access to health care, and job security [23]. The domestic work setting, typically based in isolated private households, can further hinder the reporting of abuse or workplace injuries. Live-in caregivers also perform physically demanding tasks such as lifting or assisting dependent individuals, carrying out housework, and standing for long periods. A qualitative study of migrant caregivers in Spain identified several occupational hazards, including musculoskeletal strain, falls, and injuries related to patient handling and household tasks, as well as biological risks and workplace violence [24]. At the same time, migrant live-in caregivers often face barriers such as language differences, limited knowledge of health care systems, lack of insurance, and fear of job loss, which may discourage them from reporting workplace accidents or seeking health care. In addition, studies of migrant live-in caregivers found that many workers perform tasks beyond their formal competencies, such as medical or nursing activities, without adequate training [19, 25].

Working conditions can have a substantial impact on workers’ physical and mental health. Adverse working conditions, such as long working hours, high workloads, low job control, and limited opportunities for rest, are associated with increased risks of physical complaints, sleep disturbances, cardiovascular diseases, and mental health problems including stress, anxiety, and depression [26–28]. These risks may be particularly pronounced among migrant care workers, whose employment is often characterized by precarious arrangements, extended working hours, and blurred boundaries between work and private life. Over time, such conditions may contribute to a deterioration of health. Reported health problems among migrant caregivers include physical complaints such as pain, sleep disorders, and cardiovascular issues, as well as mental health problems such as anxiety and depression [6, 17, 29–31].

Despite growing evidence on the precarious working conditions of migrant live-in caregivers, research on their occupational health and safety and their integration into occupational health systems remains scarce. To the best of our knowledge, no previous study has specifically examined live-in caregivers’ access to occupational health services, despite recent calls to address this gap [32]. For this reason, the German research project “*Live-In Health – Occupational Health Care for Temporary Migrant Workers”* was initiated. Germany provides a particularly relevant setting for this research because it has one of Europe’s largest live-in care sectors and relies heavily on migrant caregivers to meet the growing demand for home-based long-term care [11]. At the same time, the sector remains only partially regulated, creating legal uncertainty regarding employment conditions, occupational safety, and the allocation of responsibilities among caregivers, households, agencies, and public authorities. The project focused on Polish live-in caregivers because they constitute the largest national group of migrant live-in caregivers in Germany. As Polish and other Central and Eastern European caregivers typically work under comparable legal, organizational, and employment conditions, the findings are expected to be analytically transferable to other nationalities employed in the German live-in care sector. The aim of the present qualitative study was to examine the working conditions, occupational health risks, and access to occupational health care among Polish live-in caregivers in Germany. Specifically, it addressed the following research questions: (1) What work-related burdens and health risks affect Polish live-in caregivers in Germany? (2) To what extent are Polish live-in caregivers integrated into occupational health systems?

To answer these questions, we conducted qualitative interviews with live-in caregivers to explore their working conditions, knowledge of occupational health and safety, access to occupational health services, and perceived barriers and resources. As part of the broader project, interviews with occupational health stakeholders were also conducted using a comparable interview approach; these findings will be reported separately. The present article focuses exclusively on the perspectives of live-in caregivers.

## Methods

### Study population and data collection

Participants of this interview study were Polish live-in caregivers aged 18 or older who currently work or have previously worked in Germany as domestic caregivers and lived in the same household as the care recipient. Excluded were individuals from other countries and caregivers in non-live-in arrangements, such as ambulatory services.

Recruitment of live-ins was conducted nationwide using a combination of direct recruitment, key informant referrals, and snowball sampling [33]. Online resources such as websites of counseling centers for migrant workers, occupational health centers, recruitment agencies for live-ins, and social media platforms (e.g., Facebook) were used to identify potential interview partners and professionals who could facilitate contact with prospective study participants. Another recruitment channel was the German Social Accident Insurance Institution for the Health and Welfare Services (*Berufsgenossenschaft für Gesundheitsdienst und Wohlfahrtspflege* - BGW), which sent a postal letter (*n* = 50) to individuals and care services. Study information materials, e.g. flyers, were provided in both German and Polish. Suitable participants were identified and approached by the study team or contacted the team themselves after they became aware of the study. After agreeing to receive information, potential interview partners were provided with study details, data protection information, and consent forms, based on which they decided whether to participate in the study. Interviews were scheduled and conducted in Polish, the preferred language of all live-in study participants, either by phone or via video call. Returned study documents were stored in a password-protected file managed by an independent data trustee. Interview recordings (audio) were saved, anonymized during transcription, and subsequently translated into German.

Semi-structured qualitative individual interviews with Polish live-in caregivers were conducted to obtain in-depth insights into working conditions and the state of occupational health care for Polish live-in caregivers in Germany. The semi-structured interview guides were developed during qualitative research workshops, drawing on the practical knowledge and expertise of multiprofessional staff and incorporating input from the project’s advisory board, in accordance with the key principles by DeJonckheere and Vaughn [34] and Helfferich [35].

Interview guides were a priori developed in Polish and German. A pre-test in each language was conducted in advance to refine the interview guides. The interviews were held by a trained interviewer coming from a Polish-speaking family. The interviews lasted on average 50 min and focused on three main research interests: (1) work-related strain and risks of Polish live-in caregivers in Germany, (2) existing occupational health care and barriers to access, and (3) suggestions for improving occupational health provision for live-ins. An English translation of the guide is shown in the supplementary material (S1). Demographic information was collected after the interview using a short questionnaire, which is provided in the supplementary material S2.

### Data analysis

The original Polish audio recordings of the live-in-interviews were first transcribed by a native Polish speaker from the study team into pseudonymized transcripts and were then translated either by the same study member or by an externally commissioned translation agency. The German transcripts were then jointly analyzed using qualitative content analysis in MAXQDA 2024, following the inductive-deductive approach described by Mayring [36]. Initially, anticipated answer categories were derived deductively from the interview guides (e.g., work schedule, tasks). These categories were expanded inductively in an iterative process based on the interview material. Each transcript was independently coded by two members of the research team in line with the four-eyes principle. Any discrepancies were discussed until consensus was reached, ensuring consistent classification across the dataset. A condensed tree representation of the final coding system, including first- and second-level codes only, is provided in the supplementary material S3.

Risk assessment frameworks of the German care sector were applied to structure and summarize the interview data [37]. The categorization followed the occupational risk assessment approach established in Germany for workplace risk assessments (*Gefährdungsbeurteilung*) under the German Occupational Safety and Health Act (*Arbeitsschutzgesetz*). Identified work-related strains and risks were organized into four domains: (1) physical strain, (2) psychological strain, (3) occupational safety and health including working hours, and (4) exposure to biological and hazardous substances. These domains represent key areas considered in the assessment of workplace hazards and are also relevant for occupational medical prevention and surveillance (*arbeitsmedizinische Vorsorge*). Aspects related to work organization and working time are additionally considered in the German Working Time Act (*Arbeitszeitgesetz*). Although analytically distinguished, these domains are interconnected, as excessive working hours may contribute to psychological and physical strain. This framework was selected to ensure that the findings are interpretable from an occupational health perspectives.

## Results

### Demographics and work situation of the participants

A total of 16 Polish live-in caregivers participated in the study. The majority of participants were women (*n* = 15), while one participant was male. The mean age of the interviewees was 51 years, with ages ranging from 31 to 66 years. Regarding their living situation in Poland, most participants reported living alone (*n* = 11), whereas five lived with a partner, children, or other relatives. Participants had varying educational backgrounds: two reported a secondary or vocational qualification, seven had a university of applied sciences entrance qualification, four held a general higher education entrance qualification, and three had completed a university degree. Participants reported substantial experience in home care work. On average, they had worked in home care for 10 years (range: 4–25 years). Their average work experience in Germany was eight years, with a range from two to 17 years. With regard to employment arrangements, most caregivers were employed through Polish agencies (*n* = 10), while two worked via German agencies. Three participants reported being directly employed by private households, and one participant did not specify the employment model. The reported monthly net income averaged €1,650, with a range from €900 to €3,600. Participants reported lower salaries when employed through Polish agencies compared to German employers. Income further varied depending on deductions for pension and social insurance contributions, caregivers’ qualifications and negotiation capacity, as well as additional tips or informal payments provided by families.

*Let’s say the family pays €3,500, the Polish worker gets €1,500, and the German recruiter—who does nothing—takes €400. […] It looks like €3,500 or €3,000 is paid by the family—they pay different amounts, but it’s still less than a nursing home. Half, sometimes less than half, goes to the caregiver; a few hundred euros to the German coordinator; the rest to the Polish agency.* (Interview 6)

### Vocational training and prior experience

The majority of participants (*n* = 10) had completed vocational training in non-care-related fields, while four had no formal vocational training, and two had training in nursing or care. In terms of prior experience, five participants reported having provided informal care for family members, and two had completed retraining in nursing or care. Overall, one quarter of the caregivers have had a (re)training in care, one quarter had informal private caregiving experience, and one half had no prior professional experience in the field before starting work as live-in caregivers. Twelve participants began their employment in Germany with limited language proficiency (A2 level or below), while only a few (*n* = 3) started off with higher language skills (B2 and above) and one being a native German speaker.

### Motivation for taking up live-in care work

Participants described a range of personal and structural circumstances that motivated them to take up live-in caregiving positions in Germany. Eleven participants reported living alone or without family members in their household prior to employment, leaving them flexible to pursue working opportunities abroad, sometimes fueled by feelings of loneliness or a desire to leave their own living situation. Financial considerations were frequently mentioned, including income prospects and the desire to supplement their pension or to cope with financial concerns (*n* = 9). For several participants, unemployment or dissatisfaction with previous employment played a role in their decision (*n* = 7). In addition, personal contacts in Germany or recommendations from acquaintances influenced the choice to accept live-in positions (*n* = 6). Some participants referred to travel-related motivations, such as interest in the German language or the country itself (*n* = 4).

### Characteristics of care recipients and households

All care recipients were aged 80 years or older. Three respondents described caring for a couple rather than a single elderly person. Six of the interviewed live-in caregivers reported working within a rotational system, alternating with other caregivers in predefined intervals. In terms of the level of dependency, nine respondents indicated that they were caring for persons with severe impairments, such as immobility or a need for constant supervision. Six respectively seven respondents reported working with individuals who were moderately or only slightly impaired, mainly providing assistance with daily activities and household tasks. Regarding the family situation of the care recipients, nine respondents noted that family members lived nearby, whereas seven stated that the family showed little or no involvement or support in the caregiving process.

All live-in caregivers reported performing both household-related and care-related tasks. In addition, two thirds of the caregivers carried out nursing-related or medical tasks, including monitoring or assisting with medication intake, technical feeding support, applying compression stockings, wound care, insulin administration, and infusion management. Caregivers also performed organizational tasks such as coordinating appointments or communicating with service providers. The tasks performed by the live-ins are summarized in Table 1.

**Table 1:** Tasks performed by live-in caregivers (*n* = 16)

Task area	Specific activities
Household-related tasks (*n* = 16)	Cleaning, cooking, shopping
Care-related tasks (*n* = 16)	Feeding, washing, dressing, patient transfer, Physical therapy and exercise, Doctor visits
- Nursing and medical tasks (*n* = 11) (subset of care-related tasks)	Medication monitoring or assistance (*n* = 8)
	Technical feeding support (*n* = 2)
	Applying compression stockings (*n* = 2)
	Wound care (*n* = 2)
	Insulin administration (*n* = 1)
	Infusion management (*n* = 1)
Organizational tasks (*n* = 10)	Coordination of appointments, Communication with service providers

### Work-related strain and risk assessment of live-ins

The interviews revealed a wide range of occupational strains experienced by live-in caregivers, which were grouped into four main categories, following existing risk assessment frameworks as previously outlined: physical strain, psychological strain, occupational safety and health including working hours, and exposure to biological and hazardous substances.

**Physical strain** was reported primarily in connection with patient transfers, leading particularly to acute back or shoulder strain (*n* = 14). Chronic back pain (*n* = 8) was also common. Some caregivers mentioned insufficient nutritional supply during their assignments (*n* = 6), including food restrictions and rationing.

*Some families believe a caregiver is only entitled to two slices of bread per day.* (Interview 12)

**Psychological strain** emerged as a broad and complex area of concern. The most frequently mentioned issue concerned problems with the agency (*n* = 14). Live-ins reported deception and misrepresentation through incomplete information or false statements (e.g., regarding the patient’s health status, the caregiver’s qualifications communicated to clients and families, or the insurance coverage guaranteed). The participants also described poor responsiveness of agencies when problems arose, and some even reported intimidation, threats, or sanctions for alleged non-compliance with agreements that had never been documented. In addition, participants described agency communication as unclear or misleading, for example through the promotion of “24-hour care” without adequately communicating the legal boundaries of live-in caregiving work. These agency practices often resulted in organizational difficulties (see next paragraph) and fostered inappropriate expectations from clients or families (*n* = 8), which caregivers consequently had to negotiate and resist, including round-the-clock availability, extensive household labor, and the performance of medical tasks beyond caregivers’ legal scope of practice.

*That’s the worst thing about this industry: the caregivers have no one to turn to, and the agencies feel untouchable—downright brazen. As I said, when I sign up with an agency, I want to work under clear, transparent conditions. […] But I keep hearing from many caregivers about how they’re intimidated with penalties, with a whole range of issues: unpaid wages, failure to comply with agreements. Generally speaking, it’s a disaster. And that’s the biggest problem here.* (Interview 9)

*This sector operates like a grey area. I can’t even describe it. Whatever we ask for – if we ask too much – the response is: ‘Well, I guess you don’t really want to work.’* (Interview 9)

*But when it comes to mental health in the workplace, I often feel very sad and a sense of injustice. I feel very sad, frustrated, and upset that I’m not being treated the way I should be.* (Interview 7)

Additional social contributors to psychological strain included the illness or personality of the care recipient (*n* = 13), particularly dementia and conflicts with patients or relatives (*n* = 13), difficulties in language comprehension or communication (*n* = 13), feelings of dependence and obligation toward the care recipient (*n* = 11), homesickness (*n* = 11), experiences of humiliation (*n* = 9), and seclusion or isolation (*n* = 9). Feelings of fatigue and exhaustion were also common (*n* = 8). Further stressors involved dealing with death and dying (*n* = 7), emotional strain related to providing physical or emotional closeness (*n* = 7), monotony (*n* = 6), basic or restricted living conditions (*n* = 6), limited opportunities for privacy or retreat (*n* = 5), and experiences of racism or prejudice (*n* = 5). Overall, 13 participants reported psychological symptoms or diagnoses, such as grief, depression or posttraumatic stress disorder, and 9 described thoughts of or decisions to end assignments prematurely in response to psychological strain.

*It was very hard. The first few nights I cried into my pillow; I’d go into my room in the evening and just cry, thinking, “God, how hard this separation is—not seeing the kids and being in a strange apartment. It’s not my home”. It’s hard, really an enormous emotional strain*. (Interview 8)

*[…] a slave doesn’t make a good worker, do they? There are a lot of sick women with depression and various illnesses, because it’s very, very… Well, no one is capable of working seven days a week, right? Even if the patient is wonderful, the psychological strain is so terrible that there are no miracles in this line of work, is there?”* (Interview 9)

*You know what, to be honest, my health has been declining. I’ll be completely open with you—it was in [city], I was on duty. I had a patient there who was very ill, with multiple sclerosis, and he passed away. […] And I was very traumatized by it; I couldn’t cope with myself because I was so deeply connected to him, and I asked a psychiatrist for help […] I want to tell you honestly that for a long time I didn’t want to return to this profession. I was under so much stress and fear that my patient would die again.* (Interview 13)

*It’s not right that I go to bed at 11 p.m., still give out medication, get up in the middle of the night, and then have to give out medication again at 7 a.m. It’s only natural that I’ll break down eventually. I can’t take it anymore; my body is refusing to cooperate.* (Interview 7)

Regarding occupational safety, health, and working hours, caregivers most frequently reported insufficient preparation for their assignments (*n* = 16), including lacking information or training on relevant illnesses or treatments, language skills, or designated contact persons. All caregivers reported working long and irregular hours exceeding eight hours per day. A lack of free time and recovery periods (*n* = 14), as well as frequent night work (*n* = 14) were also commonly mentioned. Thirteen participants noted deviations from contractual agreements, ranging from overtime and non-contractual tasks to the use of different contracts for families and caregivers by intermediary agencies. Eleven respondents described performing medical or treatment-related nursing tasks, mostly without adequate training. Also, eleven live-ins mentioned the lack of protection in the event of liability incidents, insufficient replacement coverage and no or delayed insurance coverage in cases of personal illness. Nine live-ins reported experiences with work accidents, of whom only two received treatment in Germany through an emergency department or a designated accident insurance physician (dt. „Durchgangsarzt“). The remaining seven either did not receive any medical care or sought self-organized, self-funded treatment in Germany or Poland. Further deficits in occupational safety included unclear responsibilities and fragmented regulatory oversight between agencies, families, and caregivers (*n* = 9), insufficient accident and health protection (*n* = 6) and a lack of personal protective equipment (*n* = 5) often associated with working in private households without predefined hygiene or safety protocols, little or no professional support, and, in some cases, insufficient personal protection (*n* = 4), e.g., dealing with physical or sexual assault and privacy violations by the patient.

We didn’t have any training. I didn’t have any; I didn’t have any medical training. I didn’t have any training in how to perform patient transfers; I didn’t know the techniques for lifting or moving patients on the bed. I didn’t have the knowledge I have now. So I certainly didn’t have that back protection, and that kind of back-friendly work, that kind of ergonomically sound work. […] But they mostly hire women for the job, sometimes also nurses, but usually without nursing training, without education. So there’s no safety when it comes to lifting. As far as I know, almost no one has a lifter, so you have to lift yourself. That’s definitely a problem. (Interview 6)

*But in my first job, I worked 14 hours—no, more than that; what am I saying? Not 14 hours—from 7 a.m. to 11 p.m. Day in and day out, seven days a week, without a break, because during that time I still had to do the laundry, iron, and so on. It wasn’t that I wanted to; I just had to do it. That’s what the company had agreed upon with the client’s family*. (Interview 2)

Finally, exposure to biological and hazardous substances constituted another area of strain. Caregivers reported frequent contact with body care and excretions (*n* = 11), wound care and potential infection risks (*n* = 6), and the use of detergents and disinfectants (*n* = 6). Additionally, some participants referred to disgraceful or unhygienic living conditions (*n* = 5), such as unlocked accommodations or basement rooms with a mattress on the floor or pests.

*The fact is that our living conditions are often unacceptable. That we, for example, get a room in the basement with an old mattress whose springs stick out and hurt our back so we can’t sleep. These rooms are often dirty, full of all kinds of worms, and no one cares to disinfect them.* (Interview 5)

**Fig. 1:**
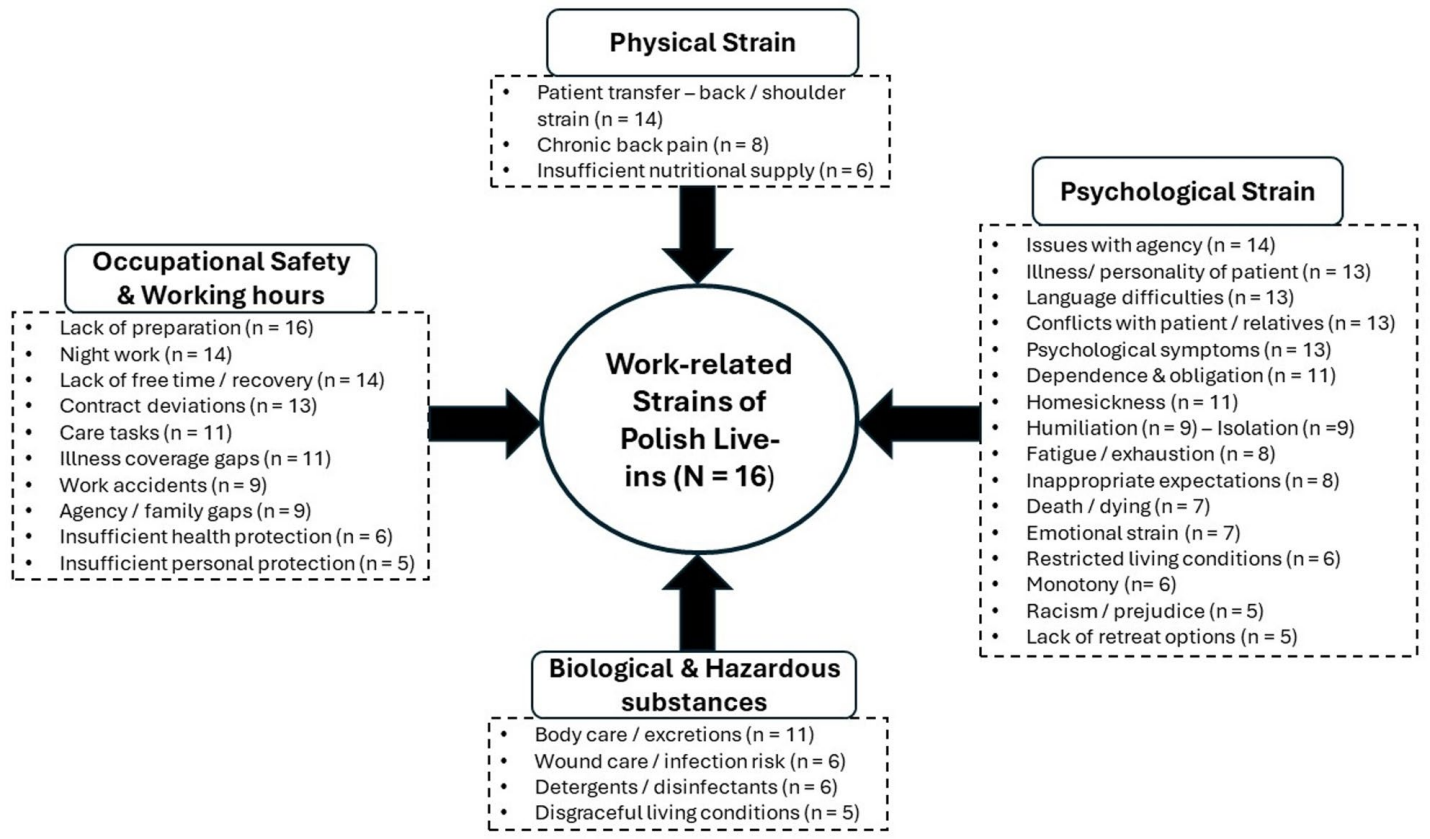
Work-related strain and risk assessment from 16 live-in interviews. The number in brackets represents those participants, who reported first-hand experiences in each subtopic

Overall, the data indicate that live-in caregivers are exposed to multiple forms of strain, encompassing physical, psychological, financial, organizational, and environmental dimensions. An overview of the identified areas of work-related strain and risk of live-ins is presented in Fig. 1.

### Integration of live-ins into occupational and general health care provision

According to the collected data, there is currently little to no systematic occupational or general health care available for migrant live-in caregivers in Germany. Insurance coverage was frequently missing, incomplete, or arranged only retrospectively after medical treatment had already become necessary (*n* = 11). Many participants experienced a diffusion of responsibility and a lack of identifiable contact persons when problems arose (*n* = 11). Access to occupational health care services was reported only once through personal contacts, while the remaining live-ins had no access to such care at all. General health care in Germany was accessed only in isolated cases of illness or accidents, when access to physicians could be facilitated through the employing family or private contacts, including linguistic support, and when private or family health insurance coverage was known (*n* = 2). Unavoidable medical contacts occurred exclusively in emergency situations via hospitals or emergency departments, even when insurance coverage was unclear (*n* = 2). In other cases of illness or accidents (*n* = 11), health care was self-organized and, in some instances, paid for privately, predominantly in Poland. Reported reasons included a preference for receiving medical treatment in a familiar language, by known physicians, and under existing insurance coverage. In some cases, placements were terminated by the employing family or agency due to incapacity to work, necessitating a return to Poland (*n* = 3).

*Well, if I have sciatica, for example, what’s the point of going to a doctor? He’ll just prescribe me some painkillers. I can bring those pills from Poland and don’t have to go to the doctor. But that won’t help me in the long run, because those pills don’t always work. […] Then rehabilitation would be necessary. But to do that, well, I wouldn’t be able to do the caretaking job, and that’s not possible, is it? So you just have to endure it and get to Poland as quickly as possible.* (Interview 15)

*Only when something happens to our health at work do they [agencies] organize the A1 certificate and send the EHIC card. […] I had a kidney problem and ended up in the hospital without an EHIC card. […] I didn’t know what would happen – would I have to pay for the hospital stay? For the medication?* (Interview 16)

Preventive and technical aids for accident prevention, such as lifters or electronic wheelchairs, were inconsistently available (*n* = 6), and also personal protective equipment such as gloves and hygienic products for personal care and wound management were, in six cases, insufficiently provided. The overall living conditions were sometimes described as inadequate (*n* = 6), including insufficient provision of food, substandard housing arrangements, violations of privacy and instances of physical violence, undermining live-ins’ sense of well-being and safety during both working hours and personal time. Liability insurance was not systematically regulated, resulting in uncertainties regarding responsibility and coverage for potential or actual property damage and personal injury.

*For example, there are all kinds of wheelchairs, walkers, and lifters because it’s very common for caregivers to have to lift residents without any assistive devices. And many caregivers end up with back problems, for example from lifting patients without having been trained to do so; they just do it by feel. […] The caregivers don’t know, they aren’t informed that in situations where the patient is bedridden—or mostly bedridden—such equipment should be available and that it is mandatory to provide it*. (Interview 12)

### Supportive and challenging work factors

Participants reported both supportive and challenging conditions in their work environment, particularly in situations where they required help. On the positive side, many reported receiving assistance and/or advice from family members of the assisted household (*n* = 15), ambulatory care staff (*n* = 13) and other live-ins, in person as well as through social media networks (*n* = 12), for example in terms of ergonomic transfer techniques. Several also relied on self-help strategies (*n* = 7), such as seeking information online or organizing their own supplies, including food, care products and medication. Although live-ins were aware of institutional points of contact, including coordinators at their respective agencies, these were often perceived as difficult to access due to limited availability, language barriers, or geographic distance (*n* = 7). Known points of contact for information or support were counseling centers for live-ins and families (*n* = 2) and social media platforms such as Facebook (*n* = 4). Some live-ins also mentioned trade unions (*n* = 1), the advisory service *Faire Mobilität* (*n* = 2), and lawyers (*n* = 2) Only two live-ins found agency coordinators helpful.

In contrast, a wide range of structural and organizational barriers were identified, resulting in unclear on-site safety conditions, unforeseeable risks, and uncertain responsibility allocation. Participants reported little to no preparation for their assignments, with few opportunities for introductory training or role-specific information (*n* = 14). They also described insufficient assessments of applicants’ suitability for particular work conditions (*n* = 11), for example when caring for patients with dementia or individuals with high body weight in settings with limited technical support.

*They take women off the street after a five-minute conversation. I feel sorry for the German families who don’t realize who’s being brought into their home. […] There aren’t any [trainings]. But they make us sign that there are. Families are assured that we had first aid training, language courses. Nonsense, it’s a lie. They take money for it, grants even, but they don’t train us. It just doesn’t exist. We’re left on our own.* (Interview 9)

Contractual arrangements were often perceived as non-transparent, vaguely formulated, or subject to deviations (*n* = 13), leaving live-ins feeling uninformed, misled, or exploited; some even reported facing unexpected threats of contractual penalties.

*Back then I didn’t know my rights at all. I didn’t know that a caregiver has to negotiate their conditions: two breaks a week, one full day off. […] The companies exploit people who don’t know what they should or shouldn’t do.* (Interview 3)

### Suggestions for improving occupational health provision for live-ins

Participants suggested several measures to strengthen occupational health provision for live-ins. They emphasized the need for transparency, training and advice in terms of relevant diseases of care recipients, their respective treatments and the usage and possible access to technical assistive devices in their designated households (*n* = 12). Theoretical and practical instruction on safe patient handling, hygiene, dealing with aggressive behavior and first aid measures were suggested:

*It’s just that I was not prepared for what I might encounter in this position – what these illnesses look like, or how to deal with the associated strain. There should be a little training on this topic, or at least some written material provided by the agencies. They know they are sending people into care work who are completely unprepared for it.*(Interview 2)

As specific formats, the participants mentioned videos, installed information boards, essential phone numbers and simple phrases to communicate typical problem scenarios in German.

Those interviewed especially stressed the importance of legal and enforceable contractual limits on working hours (*n* = 11), and proposed shift systems to reduce continuous strain caused by frequent night work and insufficient recovery periods. Improved insurance coverage was also claimed necessary (*n* = 11) – regarding personal illness (including continued payment and replacement arrangements) on the one hand and liability for potential personal or property damage on the other hand. Live-ins also called for clearer information on rights and obligations for both themselves and care-receiving families (*n* = 10). Specifically, they sought clarification that treatment-related tasks should only be carried out by appropriately trained personnel, as well as guidance on legal entitlements in home-based care, such as access to technical assistive equipment.

*The caregivers don’t know; they’re not informed that when the patient is bedridden or mostly lying down, such lifting aids should be available and must be provided. It’s unimaginable that a woman pulls a lying patient with her own strength without any device, even though such devices are normally available and easy to get.* (Interview 12)

Participants also called for stronger oversight mechanisms (*n* = 7), including preventive examinations and standardized suitability assessments by occupational physicians, minimum requirements for deployment, and monitoring of living and housing conditions. This applied both to the agencies themselves and to higher-level authorities responsible for adequate agency practices. The need for identifiable contact persons for problem situations was expressed by about a third of the sample (*n* = 5). In this context, participants mentioned both ambulatory care services for professional guidance and accessible, committed agency coordinators, advisory services, or union representatives for personal and organizational support. Personal advice was desired concerning diseases, medication and their psychological and/or physical fitness for the job. Four participants expressed the need for occupational health examinations and consultations to assure individual health support in terms of vaccinations, chronic illnesses and health prevention. Fewer suggestions referred to improved nutritional provision, language training and the development of structured peer networks among live-ins to facilitate mutual exchange and support, either in person or online. An overview of all suggestions is shown in Table 2.

**Table 2:** Suggestions for improving occupational health provision for live-ins

Area of Recommendation	Description of Suggested Measures
Theoretical and practical preparation (*n* = 12)	Transparent information, training and advice on relevant diseases of care recipients and treatments; instruction on safe patient handling, hygiene, managing aggressive behavior, and first aid, supported by videos, information boards, essential phone numbers; guidance on the use/availability of technical assistive devices.
Working hours and workload regulation (*n* = 11)	Legal and binding contractual limits on working hours; shift systems to reduce continuous strain from frequent night work and insufficient recovery periods.
Insurance coverage (*n* = 11)	Insurance regarding caregivers’ own illness (health care access, continued payment, replacement arrangements) and liability for potential damage.
Rights and obligations (*n* = 10)	Information on live-in caregivers’ rights and obligations for both caregivers and families, especially in terms of treatment-related care tasks.
Oversight and regulation (*n* = 7)	Oversight mechanisms by agencies and higher-level supervisory authorities, such as preventive examinations, standardized suitability assessments, minimum deployment requirements; monitoring of living/housing conditions.
Contact persons and advisory structures (*n* = 5)	Identifiable contact persons for problem situations; such as ambulatory care services for professional guidance, accessible agency coordinators, advisory centers, and unions for personal and organizational support.
Language support (*n* = 2)	Language training to improve communication in everyday care and emergency scenarios.
Peer support networks (*n* = 1)	Development of structured peer networks among live-ins to facilitate mutual exchange and support, in person or online.

## Discussion

The aim of this qualitative study was to explore occupational health risks and existing occupational health care for Polish live-in caregivers in Germany. The findings of 16 interviews show that live-in caregivers experience multiple, work-related strains, including physical injuries from patient handling, chronic fatigue, psychological stress from agency practices and client interactions, unsafe working conditions, and exposure to biological and hazardous substances. These strains are exacerbated by inadequate training, unclear responsibilities, insufficient protective measures, and poor living conditions, resulting in substantial physical, mental, and organizational risks. While these findings are largely consistent with previous research on migrant live-in caregivers, the present study extends the existing evidence by examining these occupational risks from an occupational health perspective and by investigating caregivers’ access to occupational health care. Overall, the results indicate that live-in caregivers constitute a particularly vulnerable group within the broader sector of migrant domestic and care work whose occupational health risks are compounded by an almost complete absence of occupational health services and preventive occupational health structures in Germany.

The vulnerability of live-in caregivers arises from a combination of structural, legal, and interpersonal factors: Many caregivers enter live-in employment under conditions shaped by economic necessity, limited employment opportunities in their countries of origin, and social circumstances such as living alone. During assignments, they both work and reside in private households, often in socially isolated settings and under temporary rotational arrangements that provide limited legal protection and few mechanisms for enforcing labor rights. These observations correspond with international evidence showing that migrant domestic workers frequently experience precarious working conditions, exploitation, and limited access to social and health protection systems [3, 17, 38, 39].

Consistent with existing international research on migrant domestic and care workers, the interviews revealed a broad spectrum of work-related strains. Physical workload was a prominent stressor. Caregivers frequently reported musculoskeletal complaints related to patient handling and mobility assistance, often performed without adequate training or access to assistive devices. These findings correspond with previous studies identifying manual lifting, patient transfers, and household tasks as key contributors to musculoskeletal disorders among migrant care workers [6, 24, 40]. Unlike institutional care, private households often lack ergonomic infrastructure, structured safety protocols, or professional support, increasing the risk of occupational injury [26, 27].

Psychological strain emerged as an equally important dimension of work-related stress. Caregivers reported stress from interpersonal challenges with care recipients and families, as well as agency-related stress, including misinformation, unclear agreements, and inadequate responsiveness in conflict situations. Similar findings have been reported in earlier studies showing that caregivers experience misinformation, miscommunication, and inadequate institutional support by intermediary agencies [6, 41]. The strong dependence on agencies and other intermediaries may further reinforce asymmetrical power relations between migrant workers, families, and recruitment structures [21, 22, 38].

Moreover, emotional strain is associated with social isolation, homesickness, language barriers, and the blurring of boundaries between work and private life inherent to live-in care arrangements. Experiences of disrespect, discrimination, or racism were also described by several interviewees. In addition, specific stressors inherent to live-in care arrangements were frequently reported, including caring for persons with dementia, communication difficulties due to limited language proficiency, and experiences of humiliation or discrimination. These findings align with international research documenting the emotional labor required in live-in care work, where caregivers must maintain close interpersonal relationships with care recipients while simultaneously coping with limited personal autonomy and restricted opportunities for social interaction [41, 42]. Previous studies similarly identify social isolation, emotional strain, and role ambiguity as common challenges among migrant domestic and care workers [17, 43]. Such conditions may result in cumulative psychological stress and contribute to mental health problems such as anxiety, depression, and burnout among migrant care workers [17, 44]. Moreover, the deterioration of health over time described by several participants reflects the broader phenomenon of the *healthy migrant effect*, which suggests that migrants initially arrive with relatively good health but may experience declining health outcomes due to structural disadvantages and cumulative occupational stressors [17, 45].

Legal and organizational uncertainty emerged as another key determinant of burden experienced by live-ins. Existing employment models, particularly the posting model and self-employment, often operate in regulatory grey zones. Within these arrangements, key elements of German labor law, including minimum wage regulations, working time limits, and safety standards, are difficult to apply or enforce in private households [18, 21, 46–48]. Although German labor standards formally apply to posted workers, in practice their implementation remains limited due to insufficient monitoring and the fragmented structure of employment relationships involving agencies, foreign employers, and private households. Caregivers may also face the risk of unintended bogus self-employment when contractual arrangements rely on informal or verbal agreements with agencies or families. These structural conditions contribute to caregivers’ reports of contractual ambiguity, irregular or excessive working hours, deviation from agreements, and insufficient protection in cases of illness or liability. Similar findings have been reported in earlier studies on live-in care arrangements in Germany and other European countries, which show that the private household as a workplace presents particular challenges for labor inspections and occupational safety enforcement [11, 21, 22]. As a result, private household as a workplace poses particular challenges for regulatory oversight, as occupational safety inspections and enforcement mechanisms are rarely applied in domestic environments and caregivers often operate in environments where occupational risks remain largely unregulated.

In addition, many participants reported misleading information prior to assignments, limited responsiveness in conflict situations, and discrepancies between contractual agreements and actual working conditions. These practices contributed to uncertainty, feelings of dependency, and difficulties in addressing workplace problems. Previous research similarly emphasizes the central role of intermediary agencies in organizing transnational care arrangements and points to the structural power asymmetries that often exist between migrant caregivers, agencies, and employing households [6, 41].

Another key finding of this study concerns the lack of access to occupational and primary health care. With only one reported instance among participants of occupational or primary care contact, and medical treatment otherwise sought privately or only in emergencies, the interviews suggest considerable gaps in the implementation of existing regulations in the field of live-in home care in Germany. Medical treatment was often sought only in emergency situations or organized privately, frequently in Poland. Similar barriers to health care access, including language difficulties, lack of information, and fears related to employment insecurity, have been reported in other studies on migrant domestic workers [17, 24]. These results align with reports from EU and international bodies calling for improved monitoring of occupational health and safety for foreign care workers, as well as limits on working hours, the enforcement of rest periods and adequate compensation [15, 49]. The inconsistent availability of accident-prevention measures and personal protective equipment further illustrates deficits in preventive occupational health structures for live-ins, especially in such physically and emotionally demanding working conditions.

Despite these structural challenges, the interviews also revealed several coping resources and supportive factors. Participants frequently reported receiving practical assistance from family members of care recipients, ambulatory care staff, and other live-in caregivers, including through informal peer networks and social media groups. These forms of informal support may help caregivers manage difficult work situations and exchange practical knowledge about caregiving tasks. Previous research has similarly emphasized the importance of social networks and peer support as coping mechanisms among migrant domestic workers [6, 30, 31]. However, while such informal resources may mitigate some challenges, they cannot compensate for structural deficiencies in occupational health protection and institutional support.

The recommendations formulated by live-in participants in this study point toward several strategies for improving occupational health provision in private households. Key suggestions included improved pre-deployment preparation and training, particularly regarding patient handling, hygiene, disease management, and communication in emergency situations. Participants also emphasized the importance of enforceable working time regulations, reliable insurance coverage, and clearer information on legal rights and responsibilities for both caregivers and employing households. In addition, caregivers called for stronger oversight of intermediary agencies and accessible contact points for advice and conflict resolution. They also highlighted regular occupational health examinations, which would already be feasible for recurring placements in the same households.

From a public health perspective, strengthening occupational health protection for live-in caregivers is not only a matter of worker protection but also a prerequisite for ensuring the sustainability and quality of home-based care in ageing societies. Across Europe, long-term care systems increasingly depend on migrant workers to meet the growing demand for care associated with demographic ageing and workforce shortages in the formal care sector [11, 50]. In Germany in particular, migrant live-in caregivers play a central role in enabling older people to remain in their own homes despite increasing care needs [18, 21]. However, this model often relies on employment arrangements characterized by limited labor protections and insufficient occupational health safeguards. Without improved regulatory frameworks, stronger enforcement of labor standards, and better access to preventive and occupational health services, the long-term sustainability of home-based care models may be at risk, both in terms of worker well-being and care quality [17, 39, 50].

### Strengths and limitations

The present study provides detailed qualitative insights into the work and occupational health situation of Polish live-in caregivers in Germany from the caregivers’ own perspectives. Conducting interviews in the participants’ native language allowed for a detailed descriptions of their experiences and facilitated open communication about sensitive topics. Furthermore, the study offered anonymous participation to ensure confidentiality and encourage live-ins to participate and to speak openly about their experiences. The diversity of participants in terms of age, work experience, and employment arrangements further contributed to a broad range of perspectives. The interviews enabled open and honest reflections on current challenges and aspects in need of change, offering a valuable basis for constructive and realistic improvements to actual working conditions in home care. Despite the small sample size typical of qualitative research, the main areas of strain and the experiences related to occupational health protection and access to services were highly consistent across participants, indicating a high degree of theoretical saturation. Moreover, while the analysis was guided by predefined deductive categories, the deductive-inductive approach also enabled the identification of additional themes and insights emerging directly from the data.

Nevertheless, several limitations should be considered when interpreting the findings. The sample size is limited to Polish caregivers, who represent the largest but not the only migrant group in this sector in Germany. The findings may therefore not be generalizable to the experiences of caregivers from other countries or working under different migration and employment conditions. As with all qualitative interview studies, the results rely on self-reported experiences and may be influenced by recall or selective reporting [51]. Despite guaranteed anonymity, it is possible that participants avoided addressing uncomfortable, socially undesirable, or potentially employment-sensitive issues. Furthermore, it cannot be ruled out that individuals in particularly precarious employment situations were less likely to participate in the study.

## Conclusions

The study highlights substantial gaps in occupational health care for Polish live-in caregivers in Germany. While the occupational risks identified largely confirm previous research on migrant live-in care, our findings reveal that these risks are accompanied by an almost complete absence of occupational health services and preventive occupational health structures. Caregivers frequently work under physically and psychologically demanding conditions with minimal preparation, limited access to preventive measures, and inconsistent insurance or medical support. Strengthening occupational health provision, through enforceable safety standards, accessible preventive services, structured training, and clear guidance on legal rights, is essential to protect this vulnerable workforce. Improving occupational health care will be essential to protect live-ins and to ensure sustainable and high-quality home-based care for the growing number of older people.

## Supplementary Information

Below is the link to the electronic supplementary material.

Supplementary Material 1

## Data Availability

No datasets were generated or analysed during the current study.

## Abbreviations

BGW: German Social Accident Insurance for the Health and Welfare Services (Berufsgenossenschaft für Gesundheitsdienst und Wohlfahrtspflege)
BMAS: German Federal Ministry of Labour and Social Affairs (Bundesministerium für Arbeit und Soziales)
EHIC: European Health Insurance Card
EU: European Union
FOGA: Research Funding Guideline on Occupational Safety and Health (Förderung der Forschung und Lehre zur Gesundheit in der Arbeitswelt)
ILO: International Labour Organization
MAXQDA: Qualitative data analysis software (VERBI Software GmbH)
SGB XI: German Social Code, Book XI (Long-Term Care Insurance) (Sozialgesetzbuch XI)
