# Supplementary material for: Occupational health risks and provision gaps among live-in caregivers: findings from a qualitative interview study of Polish live-ins in Germany
Source: J Occup Med Toxicol. 2026 Aug 7;21:24. doi: 10.1186/s12995-026-00522-8 (PMC13455274; doi:10.1186/s12995-026-00522-8)
Supplement: Supplementary file 1 [file 12995_2026_522_MOESM1_ESM.docx]

**Supplementary Material S1. Interview Guidelines – Live-ins
1. Introduction / Current work situation**

I would like to start our conversation with a few general questions…

| Guiding questions | Follow-up / Probing questions |
| --- | --- |
| How did it come about that you are working in Germany? |  |
| What did your day look like yesterday? Please start in the morning after getting up.  Was yesterday a normal working day for you? | If applicable during a free phase: please describe a typical working day instead. |
|  | What activities do you usually perform in your work? |
|  | Do you receive support from relatives or an outpatient care service? |

**2. Work context factors
2.1 Work organisation**

| Guiding questions | Follow-up / Probing questions |
| --- | --- |
| How many hours do you usually work per day? | How is your time structured? |
|  | How do you take breaks at work? |
|  | What are your rest periods like? |
|  | Do you have fixed days off and are you able to attend to personal matters during working hours? |
|  | What is the contractually agreed working time? |

**2.2 Identifying difficulties**

| Guiding questions | Follow-up / Probing questions |
| --- | --- |
| How do you feel at work? What gives you pleasure and what causes stress? | Please think of one situation that gave you great pleasure and one in which you felt overburdened. |
| How do you get along with the person you care for?  And with the relatives? | In which language do you communicate in the household? |
|  | Are there difficulties or problems at work? How do you deal with them? |
| Have you ever asked for help when you had problems at work? | What kind of help did you seek and with what outcome? |
| How do you relax and recover after a long working day? | Do you have opportunities for privacy (e.g. your own room)? |
|  | Are there any specific foods, drinks, home remedies, or medications that help you relax? |
|  | Additional aspects, e.g. leisure activities, contact with family, … |
| What work equipment and aids are available to you? What is missing? | e.g. gloves … |
| How protected do you feel at work? | e.g. Handling of needles or wounds, work equipment (e.g. gloves, face masks), occupational accidents, … |

**3. Health-related factors**

| Guiding questions | Follow-up / Probing questions |
| --- | --- |
| How is your physical and mental health?  Do you have any health problems or illnesses? | Have any conditions been diagnosed or recommendations given by a doctor? |
|  | Which complaints do you think are related to your work? |
|  | How do you cope with being separated from your family? |
| How do you take care of your health? | Do you regularly see a doctor? |
|  | Do you take any medication? If so, which? |
| What happens when you get sick?  Think about last time. | How do you deal with sickness and work? |
|  | How is healthcare organised for you in Germany (A1 certificate)? |

**4. Occupational health and safety**

| Guiding questions | Follow-up / Probing questions |
| --- | --- |
| Have you had contact with an occupational physician before or during your current assignment? What was the reason? | What did the occupational physician do (examinations, counselling)? |
|  | What did they ask you about, e.g. did they ask about former work experience/history? |
| Have you spoken to another doctor about your health (e.g. a general practitioner?) What was the reason? | What recommendations were given? |
| How were you prepared for your work in Germany?  What role did health protection play? | What offers were there to stay healthy at work (trainings, counselling, examinations, vaccinations)? |
|  | What kind of information was given to you? In what form (written, conversation…)? |
| From your point of view: What can be done to make your work safer and healthier? | What offers would you utilize or wish for (trainings, counselling, examinations, vaccinations)? |

**5. Open questions**

Guiding questions

If you could change something at your workplace, what would it be?

Have we missed anything you would like to add?

Thank you very much!

**Supplementary Material S2. Condensed code tree for the qualitative analysis of occupational health and care provision among live-in caregivers.**

Access barriers

└─ Access to physicians and limited availability

└─ Lack of information and contact points

└─ Limited language proficiency

└─ Lack of knowledge regarding entitlements and responsibilities

Characteristics of caregivers

└─ Age

└─ Motivation

└─ Requirements and skill set

└─ Language proficiency

└─ Personality

└─ Unsuitable caregivers

Characteristics of care recipients

└─ Number of care recipients

└─ Age

└─ Gender

└─ Personality

└─ Family situation

└─ Daily routine

└─ Level of dependency

└─ Illness

Health of caregivers

└─ Mental health

└─ Physical health

└─ Medical consultations

Illness of caregivers

└─ Illness as a problem at work

└─ Continuing to work while ill

└─ Work accidents

└─ Remuneration during illness

└─ Health care provision

└─ Replacement arrangements

└─ Accompaniment and support

Job satisfaction and benefits

└─ Personal aspects

└─ Social aspects

Pathways to working in Germany

└─ Family situation

└─ Income

└─ Job prospects and expectations regarding employment in Germany

└─ Personal contacts

└─ Interest in travel and language

Perceived safety

└─ Insufficient personal protection

└─ Insufficient occupational safety

└─ Organizational insecurities

└─ Protective equipment
(e.g. disinfectants, gloves)

Preparation for caregiving work

└─ Lack of preparation

└─ Preparation in Poland / Polish agencies

└─ Preparation in Germany / German agencies or intermediaries

└─ Individual initiative

Professional background

└─ No formal caregiving training / learning by doing

└─ Non-care-related professional experience

└─ Professional caregiving experience

Relationships

└─ With care recipients

└─ With relatives

└─ With colleagues

└─ With physicians and clinical staff

Support options

└─ Relatives and family of the care recipient

└─ Other caregivers

└─ Own family and friends

└─ Neighbourhood

└─ Internet-based support

└─ Support provided by agencies

└─ Professional nursing support

└─ Professional assistance with daily living

└─ Professional medical care

└─ Other formal support options
(e.g. lawyers, trade unions)

└─ Lack of support

└─ Use of support services

Tasks performed

└─ Household-related tasks

└─ Organizational tasks

└─ Care-related tasks

└─ Medical tasks

Work equipment and aids

└─ Technical aids

└─ Non-technical aids and utensils

└─ Needs

└─ Budget for work equipment and aids

Work–life balance

└─ Duration of assignments

└─ Housing situation, opportunities for retreat

└─ Provision of food and medication

└─ Leisure activities

└─ Personal contacts

└─ Basic or restricted living conditions

└─ Inadequate or undignified living conditions

Work-related dissatisfaction and strain

└─ Work-related factors

└─ Problems with agencies

└─ Problems with care recipients/families

└─ Social factors

└─ Environmental and situational factors

Work situation

└─ Organization

└─ Remuneration

└─ Contractual arrangements

Working hours

└─ Number of working hours per day/week

└─ Duration of assignments

└─ Night work

└─ Breaks

└─ Rest days and leave

└─ Termination of assignments

Workplace improvement

└─ Work organization

└─ Improved basic provision (budget, food)

└─ Remuneration

└─ Insurance coverage

└─ Information

└─ Training

└─ Counselling

└─ Occupational medical examinations

└─ Support by agencies

└─ Other support services

└─ Appreciation and respe

**Supplementary Material S3. Short Questionnaire – Live-ins**
**General Questions About You and Your Occupation**

A1. What is your gender?

☐ Female

☐ Male

☐ Diverse / non-binary

A2. How old are you? ______ years

A3. What is your marital status?

☐ Married

☐ Single

☐ Divorced

☐ Widowed

☐ Other: ____________________

A4. Do you live in Poland alone or together with other people in a shared household? (Multiple answers possible)

☐ Alone

☐ With partner / spouse

☐ With child(ren)

☐ With other relatives

☐ With other persons

A5. How many children do you have? ______ child(ren)

A6. What is the highest level of general education you have completed?

☐ Primary school certificate

☐ Lower secondary school certificate (vocational)

☐ Upper secondary vocational qualification

☐ General higher education entrance qualification

☐ Other: ____________________

A7. What profession did you train for? ____________________

A7a. I have not completed any vocational training. ☐ Yes

A8. How many years of experience do you have in caregiving? ______ months ______ years

A9. For how many months or years have you been working as a caregiver in Germany? ______ months ______ years

A10. How many people have you cared for so far in a home-based setting? ____________________

**Current Employment Situation**

A11. Are you currently on assignment or in a break period?

☐ On assignment – How long has your current assignment lasted so far? ____________________

☐ Break period – How long ago did your last assignment end? ____________________

A12. Who exactly is your employer?

☐ The household (or relatives) is/are the employer(s).

☐ A company is the employer – Country: ☐ Germany ☐ Poland ☐ Other: ____________

☐ I am a self-employed caregiver.

☐ It is organized differently: ____________________

A13. What is your monthly net income?

☐ Below €520

☐ €521 to €900

☐ €901 to under €1,300

☐ €1,300 to under €1,500

☐ €1,500 to under €2,000

☐ €2,000 to under €2,600

☐ €2,600 to under €3,600

☐ €3,600 to under €5,000

☐ Don’t know

☐ No answer	Thank you very much for your participation!
